# Renal artery stenosis presenting as preeclampsia

**DOI:** 10.1186/s40885-020-00140-4

**Published:** 2020-04-01

**Authors:** Michael Brandon Omar, William Kogler, Satish Maharaj, Win Aung

**Affiliations:** 1 Department of Medicine, University of Florida College of Medicine-Jacksonville, Jacksonville, USA; 2 Division of Medical Oncology and Hematology, University of Louisville, Louisville, USA

**Keywords:** Preeclampsia, Renal artery stenosis, Renovascular hypertension, Secondary hypertension

## Abstract

**Background:**

Renal artery stenosis is a notorious cause of secondary hypertension which classically presents as chronic refractory hypertension, recurrent flash pulmonary edema or renal insufficiency after initiation of an angiotensin converting enzyme inhibitor. Rarely, there have been reported cases of pregnant patients presenting with new onset or superimposed preeclampsia secondary to renovascular hypertension. In this subset of patients, renovascular hypertension carries significantly higher risks including obstetric, fetal and medical emergencies and death. Prompt treatment is required. However, the teratogenic risks of radiological investigations and antihypertensive medications limit diagnostic and management options thus posing quite a dilemma.

**Case presentation:**

A 38-year-old female, at 33 weeks of gestation, was hospitalized for preeclampsia with severe features. A viable neonate had been expeditiously delivered yet the patient’s post-partum blood pressures remained severely elevated despite multi-class anti-hypertensive therapy. Renal artery dopplers revealed greater than 60% stenosis of the proximal left renal artery and at least 60% stenosis of the right renal artery. Renal angiography showed 50% stenosis of the left proximal renal artery for which balloon angioplasty and stenting was performed. The right renal artery demonstrated less than 50% stenosis with an insignificant hemodynamic gradient, thus was not stented. Following revascularization, the patient’s blood pressure improved within 48 h, on dual oral antihypertensive therapy.

**Conclusions:**

Preeclampsia that is refractory to multi-drug antihypertensive therapy should raise suspicion for renal artery stenosis. Suspected patients can be screened safely with Doppler ultrasonography which can be then followed by angiography. Even if renal artery stenosis does not seem severe, early renal revascularization may be considered in patients with severe preeclampsia who do not respond to antihypertensive management.

## Background

Renal artery stenosis is a notorious cause of secondary hypertension resulting from the activation of the renin-angiotensin system in response to reduced renal blood flow. Classic presentations include chronic refractory hypertension, recurrent flash pulmonary edema and renal insufficiency after initiation of an angiotensin converting enzyme inhibitor. Although rare, there have also been reported cases of pregnant patients presenting with new onset or superimposed preeclampsia secondary to renovascular hypertension [1, 2]. In this subset of patients, renovascuar hypertension carries significantly higher risks including obstetric, fetal and medical emergencies and death. Prompt treatment is required. However, the teratogenic risks of radiological investigations and antihypertensive medications such as angiotensin converting enzyme inhibitors or aldosterone antagonists limit management options and poses quite the dilemma. When possible, expedited delivery is beneficial; notwithstanding the fact that there has been success with interventional treatment prior to successful delivery. Furthermore, even after delivery, the mortality risk of pre-eclampsia continues into the post-partum period thus urgent and aggressive treatment strategies should continue to be pursued for these patients including consideration of early revascularization.

## Case presentation

**Fig. 1:**
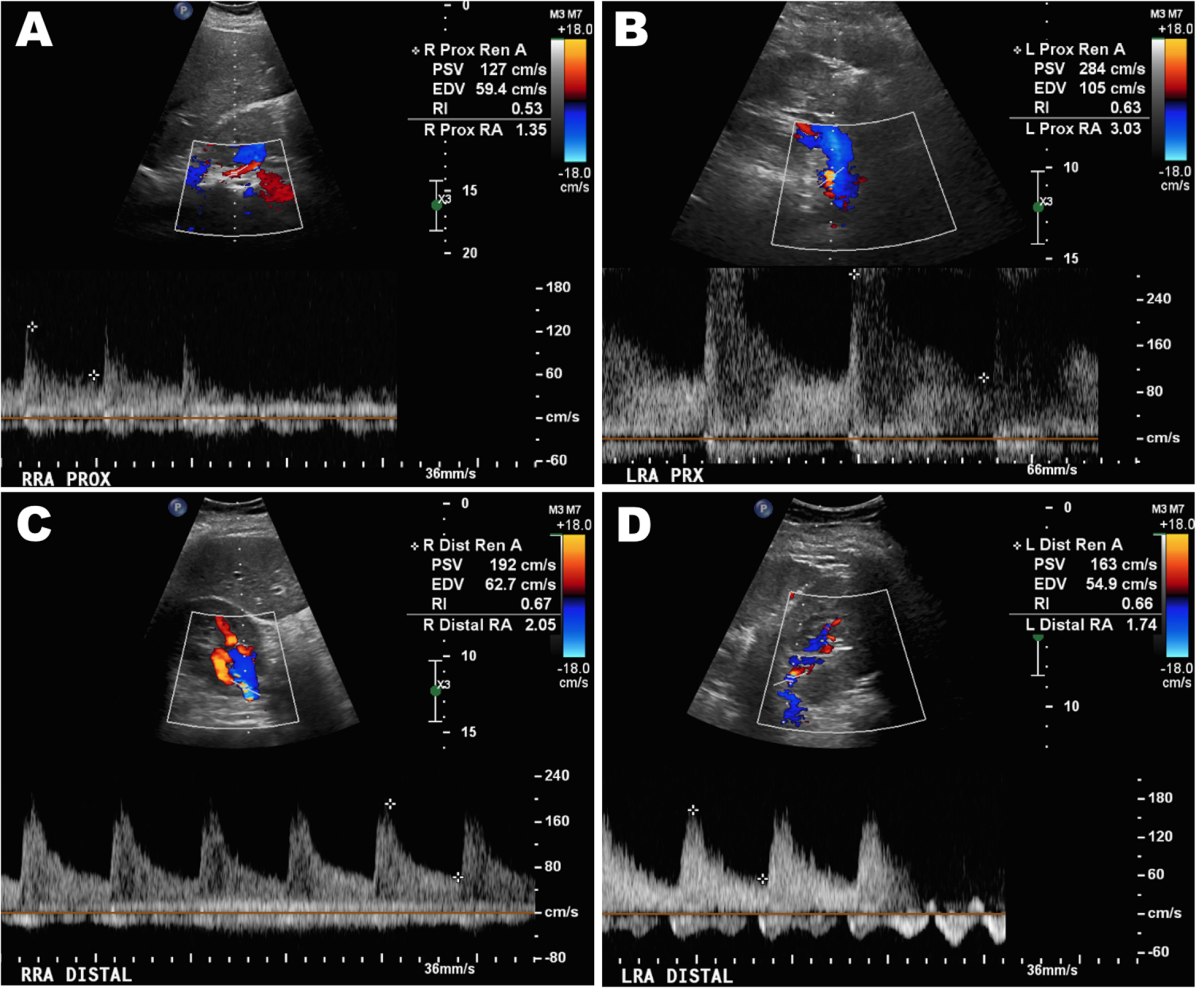
Doppler ultrasonography with peak systolic velocities (PSV) of the right proximal (**a**), left proximal (**b**), right distal (**c**) and left distal (**d**) renal arteries [Normal PSV < 180 cm/s]

**Fig. 2:**
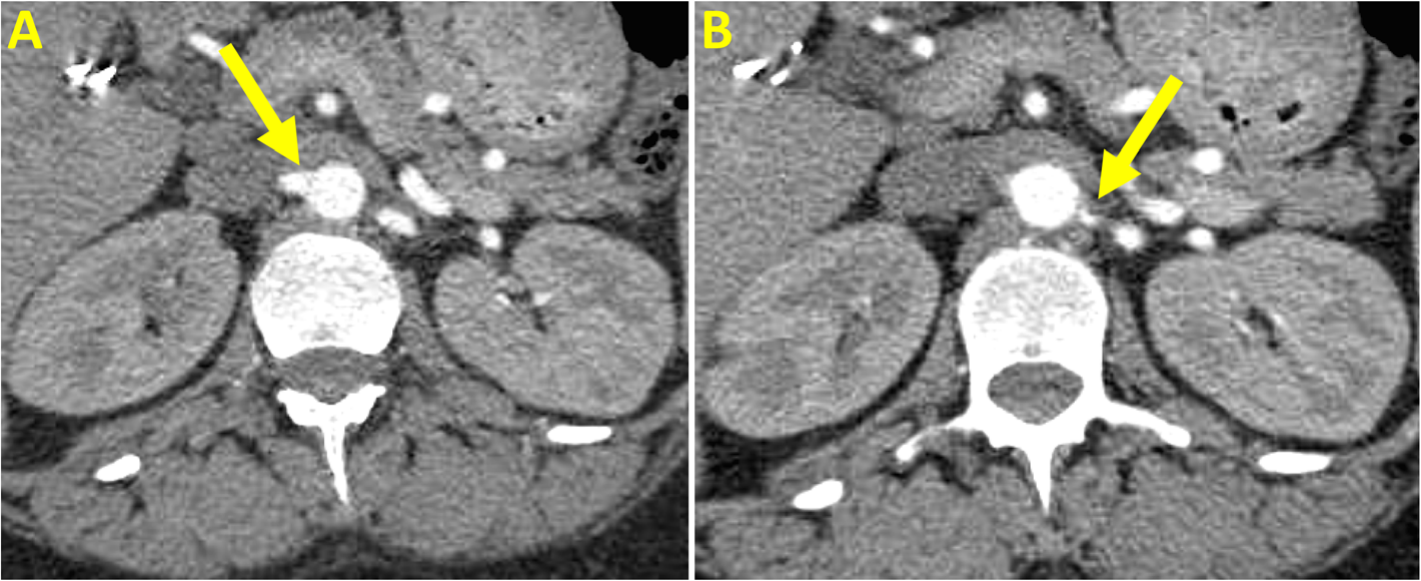
Computerized tomography of the right (**a**) and left (**b**) proximal renal arteries (arrows). Approximately 50% stenosis of the left renal artery is noted

**Fig. 3:**
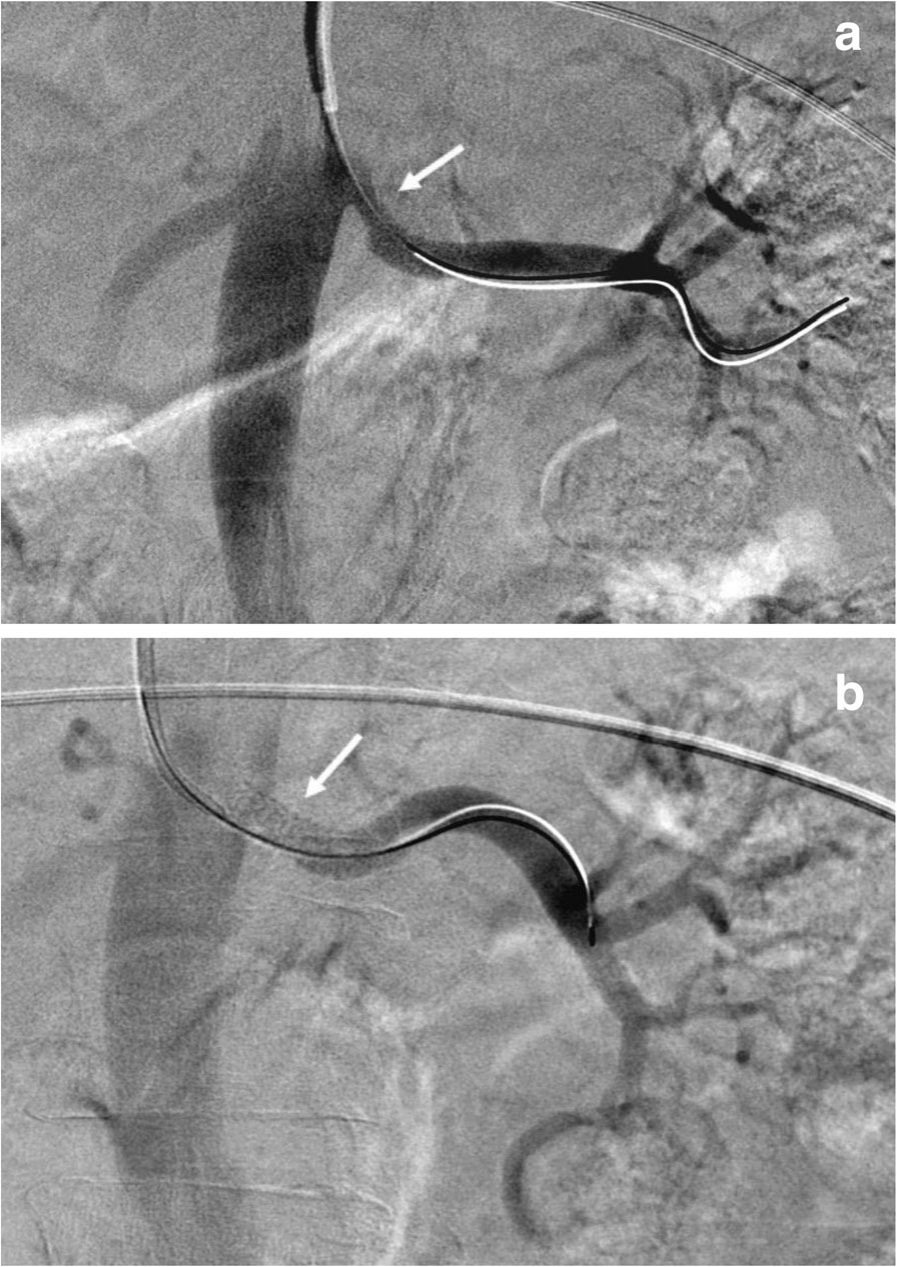
Percutaneous renal angiography showing the proximal left renal artery (arrows) with 50% stenosis prior to stent placement (**a**) and improved flow post stent placement (**b**)

A 38-year-old female, gravida 3 para 2 at 33 weeks of gestation, was hospitalized for preeclampsia with severe features. A viable neonate had been expeditiously delivered yet the patient’s post-partum blood pressures remained severely elevated ranging from 230/130 mmHg to 280/170 mmHg. She had no antenatal care but reported a history of uncomplicated hypertension during her prior pregnancies and tobacco abuse which was stopped 8 months prior. At the bedside, she complained of mild headaches but denied visual disturbances or upper abdominal pain. She was alert and well oriented with a pulse of 80 bpm. There was no hyperreflexia, clonus, papilledema, peripheral edema or signs of pulmonary edema. Her examination was otherwise unremarkable including the absence of renal bruits. Apart from an elevated random urine protein to creatinine ratio of 0.7, the laboratory investigations were within normal limits including serum creatinine, electrolytes, platelet count, liver function and coagulation studies. There were no laboratory features of hemolysis. She was treated with multiple anti-hypertensives over the next 72 h including oral nifedipine, labetalol and clonidine as well as intravenous infusions of labetalol, nicardipine, hydralazine. Magnesium was used for eclampsia prophylaxis. Of note, a single dose of intravenous enalapril was given with a subsequent 60% increase in serum creatinine that returned to baseline within 24 h of discontinuation. Renal artery dopplers (Fig. 1) were performed which revealed greater than 60% stenosis of the proximal left renal artery and at least 60% stenosis of the distal right renal artery. Computerized tomography angiography showed approximately 50% stenosis of the proximal left renal artery without stenosis of the right renal artery (Fig. 2). At this juncture, in the setting of recalcitrant severe preeclampsia and the mortality risk of impending eclampsia, an invasive strategy for better evaluation and possible intervention was deemed net beneficial. Renal angiography showed 50% stenosis of the left proximal renal artery for which balloon angioplasty and stenting was performed (Fig. 3). The right renal artery demonstrated less than 50% stenosis with an insignificant hemodynamic gradient, thus was not stented. Following revascularization, the patient’s blood pressure improved, ranging from 180/100 mmHg to 160/90 mmHg within 48 h, on dual oral antihypertensive therapy. She was ultimately discharged to titrate further anti-hypertensive therapy as an outpatient.

## Discussion

Renal artery stenosis is a well-established cause of secondary hypertension resulting from the activation of the renin-angiotensin system in response to reduced renal blood flow. Atherosclerosis is the most common etiology and is usually suspected in patients over the age of 45, dyslipidemic patients, or smokers. However, other etiologies such as fibromuscular dysplasia in younger patients or Takayasu’s arteritis should be considered. Atherosclerotic stenosis typically affects the proximal main renal artery near the ostium compared to fibromuscular dysplasia which typically affects the distal segments.

Classic presentations include chronic refractory hypertension, recurrent flash pulmonary edema and renal insufficiency- notably after initiating an angiotensin converting enzyme inhibitor (ACE-I) or angiotensin receptor blocker (ARB). Although rare, there have also been reported cases of pregnant patients presenting with new onset or superimposed preeclampsia secondary to renovascular hypertension [1, 2].

Of the different modalities used to investigate renal artery stenosis, doppler ultrasonography is the safest and has a sensitivity of at least 85%, though it frequently overestimates stenoses as in our case [3, 4]. Magnetic resonance or computerized tomography angiography have superior diagnostic accuracy with a sensitivity of 94% but the gold standard remains conventional catheter based angiography [5]. Supplemental studies such as direct renal vein renin, captopril renography or plasma renin activity to aldosterone ratios may be helpful in diagnostic dilemmas, though not currently routinely recommended [6].

Treatment may involve aggressive medical therapy with statins, antiplatelets and antihypertensives and/or renal artery revascularization. Historically, ACE-I or ARB therapy has been cautioned especially in bilateral renal artery stenosis because of the possibility of reduced post-stenotic renal perfusion pressures and subsequent ischemic nephropathy and renal failure. However, there have been observational studies suggesting a mortality benefit to closely monitored ACE-I or ARB treatment [7].

In terms of invasive treatment, percutaneous transluminal renal angioplasty with or without stenting has become the standard versus surgical revascularization. Although a recent systematic review showed only marginal benefit to this approach compared to medical therapy alone, there is evidence that select patient do have significant benefits in blood pressure control [2, 8, 9]. Furthermore, studies have shown that usually at least 80% stenosis is required to produce any significant hemodynamic stimulus to the renin-angiotensin system and thus may be a threshold for invasive treatment [10, 11]. However, as in our case, few patients have been shown to benefit from revascularization at stenoses of as low as 50% [8, 11]. Additionally, these hemodynamic studies were performed in non-pregnant patients. Thus, whilst these data are important to avoid unnecessary procedures, clinical acumen remains necessary for select cases where revascularization of seemingly insignificant stenoses may yet produce a clinical response.

In women with preeclampsia due to renovascular hypertension, there is significant risk for obstetric and medical complications including death especially with severe preeclampsia (blood pressures more than or equal to 160/90 mmHg even without signs of end organ dysfunction or hemolysis). Prompt treatment is required. Yet, the teratogenic risks of radiological investigations and antihypertensive medications such as ACE-I/ARB’s limit diagnostic and management options and pose quite a dilemma. When possible, expedited delivery is beneficial. However, there has been some success with interventional treatment prior to delivery [2]. The high mortality risk of eclampsia continues into the the post-partum period and it is uncertain when blood pressures can be expected to normalize in preeclampsia even in the absence of renovascular hypertension [12]. Therefore, an urgent and aggressive management strategy should be pursued for these patients with consideration for early revascularization if a rapid clinical response is not seen with medical management.

## Conclusions

Preeclampsia that is refractory to multi-drug antihypertensive therapy should raise suspicion for renal artery stenosis. Suspected patients can be screened safely with Doppler ultrasonography which can be then followed by angiography. Even if renal artery stenosis does not seem severe, early renal revascularization may be considered in patients with severe preeclampsia who do not respond to antihypertensive management.

## Data Availability

Not applicable.

## Abbreviations

ACE-I: Angiotensin converting enzyme inhibitor
ARB: Angiotensin receptor blocker
